# Genetic Evolution and Implications of the Mitochondrial Genomes of Two Newly Identified *Taenia* spp. in Rodents From Qinghai-Tibet Plateau

**DOI:** 10.3389/fmicb.2021.647119

**Published:** 2021-03-23

**Authors:** Yao-Dong Wu, Li Li, Yan-Lei Fan, Xing-Wei Ni, John Asekhaen Ohiolei, Wen-Hui Li, Jian-Qiu Li, Nian-Zhang Zhang, Bao-Quan Fu, Hong-Bin Yan, Wan-Zhong Jia

**Affiliations:** ^1^State Key Laboratory of Veterinary Etiological Biology, National Professional Laboratory for Animal Echinococcosis, Key Laboratory of Veterinary Parasitology of Gansu Province, Lanzhou Veterinary Research Institute, Chinese Academy of Agricultural Sciences, Lanzhou, China; ^2^School of Pharmaceutical Sciences, Tsinghua University, Beijing, China; ^3^Animal Disease Prevention and Control Center of Guizhou Province, Guiyang, China; ^4^Jiangsu Co-innovation Center for Prevention and Control of Important Animal Infectious Disease, Yangzhou, China

**Keywords:** mtDNA, Qinghai-Tibet Plateau, phylogeny, divergence time, *Taenia* spp.

## Abstract

The larva of Taeniidae species can infect a wide range of mammals, causing major public health and food safety hazards worldwide. The Qinghai-Tibet Plateau (QTP), a biodiversity hotspot, is home to many species of rodents, which act as the critical intermediate hosts of many Taeniidae species. In this study, we identified two new larvae of *Taenia* spp., named *T. caixuepengi* and *T. tianguangfui*, collected from the plateau pika (*Ochotona curzoniae*) and the Qinghai vole (*Neodon fuscus*), respectively, in QTP, and their mitochondrial genomes were sequenced and annotated. Phylogenetic trees based on the mitochondrial genome showed that *T. caixuepengi* has the closest genetic relationship with *T. pisiformis*, while *T. tianguangfui* was contained in a monophyletic group with *T. crassiceps*, *T. twitchelli*, and *T. martis*. Biogeographic scenarios analysis based on split time speculated that the speciation of *T. caixuepengi* (∼5.49 Mya) is due to host switching caused by the evolution of its intermediate host. Although the reason for *T. tianguangfui* (∼13.11 Mya) speciation is not clear, the analysis suggests that it should be infective to a variety of other rodents following the evolutionary divergence time of its intermediate host and the range of intermediate hosts of its genetically close species. This study confirms the species diversity of Taeniidae in the QTP, and speculates that the uplift of the QTP has not only a profound impact on the biodiversity of plants and animals, but also that of parasites.

## Introduction

The most recent molecular phylogenetic analysis has suggested that the family Taeniidae (Eucestoda: Cyclophyllidea) should be composed of four genera: *Taenia*, *Echinococcus*, *Hydatigera*, and *Versteria* (Nakao et al., 2013). Among them, *Taenia* and *Echinococcus* species pose a serious public health threat to humans and animals globally. Terrestrial mammals are crucial to the life cycle of taeniids. Most adult tapeworms parasitize the intestines of carnivores while the intermediate hosts harbor the larva stage that develops from ingested eggs, causing severe health effects (Jia et al., 2012; Nakao et al., 2013; Lymbery, 2017; Deplazes et al., 2019).

Before the new classification recommendation of Nakao et al. (2013), two genera (*Taenia* and *Echinococcus*) were generally accepted. *Taenia* was constituted by about 42 valid species and three subspecies based on morphology (Hoberg et al., 2000; Hoberg, 2006; Nakao et al., 2013). As for *Echinococcus*, a total of 16 species and 13 subspecies were described based on morphology, but most of these taxa were subsequently invalidated following widespread application of molecular genetic methods (Lymbery, 2017). It is difficult to distinguish taeniid species according to their morphological characteristics at different stages of their life cycle, even by specialists (Flisser et al., 2005; Mathis and Deplazes, 2006; Jia et al., 2012). Sometimes, morphological characteristics are substantially influenced by the different intermediate host origins (Lymbery, 1998).

Mitochondrial (mt) DNA sequence has been recognized among the most suitable molecular markers of molecular ecology, population genetics, evolutionary biology and biological differentiation due to its high mutation rate and maternal inheritance (Hebert and Gregory, 2005; Will et al., 2005; Hajibabaei et al., 2007; Jia et al., 2012). In the last two decades, comparative analyses of taeniid mtDNAs have been increasingly applied in phylogenetic studies, for inferring evolutionary relationship, new species identification, species reclassification, phylogeography, genetic diversity, and tracing of evolutionary origins of related and identical species (Xiao et al., 2005; Nakao et al., 2007; Nakao et al., 2013; Terefe et al., 2014; Kinkar et al., 2018). Among the taeniid family, mt genomes of 36 species and genotypes have been sequenced and are available on GenBank^1^, providing valuable data support for phylogenetic studies of Taeniidae.

The shrinkage and fragmentation of wildlife habitats due to human activities can lead to increased contact between humans or livestock and wildlife, which potentially increases the risk of transmission of natural focal disease (Suzán et al., 2008). Rodents, the largest (∼43% of all mammal species) and most widely distributed group of mammals, act as major vectors of human and domestic animal diseases (Singla et al., 2008; Wu et al., 2018). The Qinghai-Tibet Plateau (QTP), one of the biodiversity hotspots on earth, is habitat to a rich diversity of wild rodent species (Zhou and Ma, 2002), as well as many rodent-eating carnivores (Smith et al., 2019), creating the conditions for various taeniid species to complete their life cycles. The high altitude geographic isolation combined with the geological complexity of the QTP increases the opportunities for genetic variation and speciation, leading to the continuous discovery of new species of rodents and Taeniidae (Xiao et al., 2005; Dahal et al., 2017). However, few studies have involved the population structure and biodiversity of taeniid species in QTP, except for *Echinococcus*.

As endangered or protected carnivores are difficult to sample, we collected metacestode samples of rodents to investigate the biodiversity and distribution of taeniid species in QTP. In this study, two new mt genomes of the metacestode samples were firstly sequenced and annotated. Through the phylogenetic analysis of mt genomes with species in the four different genera of taeniids, the validity of these two new *Taenia* spp., named *T. caixuepengi* and *T. tianguangfui* larvae, were confirmed and their phylogenetic relationship and evolutionary origin were analyzed.

## Materials and Methods

### Ethics Statement

All animals were handled in strict accordance with good animal practice according to the Animal Ethics Procedures and Guidelines of the People’s Republic of China, and the study was approved by the Animal Ethics Committee of Lanzhou Veterinary Research Institute, Chinese Academy of Agricultural Sciences (No. LVRIAEC2012-007).

### Parasite Materials

Plateau pikas (*Ochotona curzoniae*) and Qinghai voles (*Neodon fuscus*) were trapped in Darlag county (33°43′N; 99°38′E; altitude at 4,068 m) and Jiuzhi county (33°19′N; 100°32′E; altitude at 3,832 m) of Qinghai province, the People’s Republic of China in July 2013. Following ethical approval, all trapped pikas and voles were dissected regarding the enterocoelia, chest and cranial cavities. Many banded cysticerci were collected in the enterocoelia of Plateau pika (Supplementary Figures S1A,B) and numerous lenticular cysticerci were collected in the enterocoelia and chest of Qinghai vole (Supplementary Figures S1C,D). Detailed sample collection data can be found in Supplementary Table S1. After detaching the lesions, the cysticerci were put into 75% (v/v) ethanol for molecular and morphological identification. Cysticerci from pikas and voles were photographed by Thermo Scientific^TM^ Apreo S SEM, and their hooks were hand-drawn using Point 3D of Microsoft.

### DNA Isolation, Amplification, and Sequencing

*Cysticercus* DNA was extracted using a commercial kit as instructed by the manufacturer (Blood and Tissue Kit, Qiagen, Germany). The mt genomes of *Taenia* spp., whose intermediate hosts include rodents, downloaded from GenBank (Supplementary Table S3) were aligned by using MEGA 7.0. Nine overlapping primers targeting the complete mt genome were designed using Oligo 6.0 at relatively conserve regions observed on alignment of the mt genome sequences. The primer sequences (Supplementary Table S2) were synthesized by Genewiz Biotech (Beijing, China). A standard 50 μl PCR protocol was used to amplify the mtDNA fragments. PCR products were purified directly from an agarose gel (1%) using an Axy Prep^TM^ DNA Gel Extraction Kit (AXVGEN, United States) and then sent to the company Genewiz Biotech for sequencing.

### Mitochondrial Genome Annotation

These two mtDNAs were assembled manually, and annotated preliminarily by Geseq^2^ with the reference of related species, *T. pisiformis* and *T. crassiceps*, identified by the *cox*1 gene alignment of Neighbor-Joining method in MEGA 7.0 (data not shown). Putative tRNA genes were identified using ARWEN^3^ (Laslett and Canbäck, 2008). The positions of their open reading frames and rRNA genes were also further checked and modified based on alignment with the mt genomes annotation of *T. pisiformis* and *T. crassiceps*, respectively. SnapGene (v3.2.1) was used to translate the amino acid sequence of the protein-coding genes with Echinoderm, Flatworm Mitochondrial genetic code and map the annular diagram of the mt genomes.

### Phylogenetic Analyses

To determine the phylogenetic status of these two Taenia spp., the phylogenetic trees were constructed using Bayesian methods in MrBayes v3 with the tandem DNA sequences and amino acid sequences of 12 encoding genes in their mt genomes and other 32 taeniid mt genome sequences downloaded from GenBank, while the sequences of *Schistosoma japonicum* was used as outgroup (Supplementary Table S2). For the amino acid data set, the mixed model was applied (prset aamodelpr = mixed); two chains (temp = 0.2) were run for 3,000,000 generations and sampled every 1,000 generations (Rota-Stabelli et al., 2009). For the nucleotide data set, Modeltest 3.7 maxX (Posada and Crandall, 1998) was used to estimate a suitable model for nucleotide substitution; this was equivalent to GTR + I + G and settings were nst = 6, rates = invgamma, ngammacat = 4. Four chains (temp = 0.2) were run for 1,000,000 generations and sampled every 1,000 generations. The first 25% of trees were omitted as burn-in and the remaining trees were used to calculate Bayesian posterior probabilities. The best Bayesian tree was then compiled and processed by FigTree.v1.4.4.

### Divergence Times Analysis

The phylogenetic trees were used as a reference for species selection in divergence times analysis. *Echinococcus multilocularis* and *E. shiquicus* were also selected because the parasitism of their larvae is also found in Plateau pika (*O. curzoniae*) (Wang et al., 2018), besides *E. multilocularis* is the sister species of *E. shiquicus* (Lymbery, 2017). Divergence times were calculated from the concatenated CDS alignment of the 12 mitochondrial protein-coding genes by BEAST2 (v2.6.2). The Strict Clock model was chosen to ignore the rate differences between the branches in the mode. The gamma category count was set to 4, and HKY substitution model was selected with the empirical setting from the frequencies in site model. Other settings, such as substitution rate and shape, in the site model were evaluated in the analysis. The calibrated Yule model was used as the tree prior. Time calibration was calibrated with the previously estimated date between *T. saginata* and *T. asiatica* (∼1.14 Mya) (Michelet and Dauga, 2012; Wang et al., 2016). Samples from the posterior were drawn every 1,000 steps over a total of 10,000,000 steps per MCMC run. Other options were run on their default values. The convergence of likelihood values was determined by Tracer (v1.7.1). Trees were annotated by TreeAnnotator (v2.1.2) using maximum clade credibility tree and median heights settings with 50% burn-in. The evolutionary divergence time of the intermediate host, Qinghai vole (*N. fuscus*), was also calculated with the concatenated CDS alignment of 13 mt protein-coding genes of the rodents, and the species involved were selected from our previous report (Li et al., 2019; Supplementary Table S3). The time calibration was based on the divergence time of *Mus* and *Rattus* (11–13 Mya) (Wang et al., 2020), and other parameters were the same as above.

## Results

### General Features of the Mitochondrial Genome of Two Parasites

**FIGURE 1:**
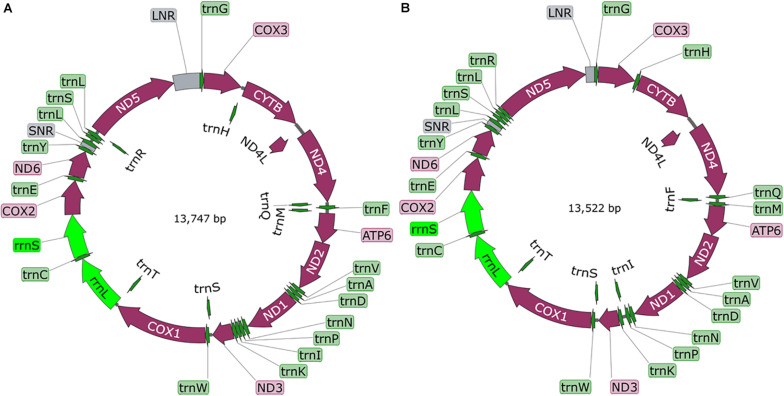
The diagram of complete mitochondrial genome of *Taenia tianguangfui* **(A)** and *Taenia caixuepengi* **(B)**. The protein-encoding genes are depicted in plum, the tRNAs are depicted in green, the rRNAs are depicted in light green and the non-coding mitochondrial regions (NCRs including LNR and SNR) are depicted in gray. The inferred gene boundaries of them are shown in Table 1.

A total of 300 pika (125) and voles (175) were examined. Overall, 7.3% were infected with cysticerci (see Supplementary Table S1 for more details). Amplification and sequencing of a fragment of the *cox*1 gene using the conserved JB3 and JB4.5 primers (Bowles et al., 1992) confirmed their identity. From both study sites, cysticerci from pikas were the same new species, named *T. caixuepengi* larva, meanwhile, the cysticerci from voles were morphologically different from *T. caixuepengi* larva and were named *T. tianguangfui* larva. Their complete mt genomes were sequenced and spliced, and were 13,747 bp (GenBank ID: MT882036) and 13,522 bp (GenBank ID: MT882037) in length, respectively. Both contain 2 rRNA genes [the small (*rrn*S) and large (*rrn*L) subunits of rRNA], 12 protein-encoding genes (*atp*6, *cyt*b, *nad*4L, *cox*1-3, and *nad*1-6) and 22 tRNA genes, but lack *atp*8 gene, which are typical of cestode mt genomes (Figure 1). The inferred gene boundaries and their lengths are shown in Table 1.

**TABLE 1:** Positions and gene lengths in the mitochondrial genomes of *Taenia tianguangfui* (Tt), *T. caixuepengi* (Tc).

Genes	Positions (length, bp)		Initiation and termination codons		Anticodons
	Tt	Tc	Tt	Tc	
*trn*G	1–65 (65)	1–68 (68)			TCC
*cox*3	68–715 (648)	72–722 (651)	ATG/TAG	GTG/TAA	
*trn*H	724–792 (69)	716–786 (71)			GTG
*Cyt*b	796–1,863 (1,068)	790–1,857 (1,068)	ATG/TAG	ATG/TAA	
*nad*4L	1,865–2,125 (261)	1,857–2,117 (261)	ATG/TAG	ATG/TAA	
*nad*4	2,092–3,348 (1,257)	2,084–3,337 (1,254)	ATG/TAG	ATG/TAA	
*trn*Q	3,349–3,409 (61)	3,338–3,400 (63)			TTG
*trn*F	3,409–3,472 (64)	3,400–3,463 (64)			GAA
*trn*M	3,469–3,534 (66)	3,461–3,524 (64)			CAT
*atp*6	3,535–4,053 (519)	3,531–4,043 (513)	ATG/TAG	ATT/TAA	
*nad*2	4,058–4,930 (873)	4,045–4,917 (873)	ATG/TAA	ATG/TAG	
*trn*V	4,947–5,011 (65)	4,922–4,983 (62)			TAC
*trn*A	5,012–5,075 (64)	4,996–5,058 (63)			TGC
*trn*D	5,085–5,149 (65)	5,067–5,128 (62)			GTC
*nad*1	5,154–6,047 (894)	5,134–6,030 (897)	ATG/TAA	ATG/TAA	
*trn*N	6,064–6,131 (68)	6,044–6,109 (66)			GTT
*trn*P	6,141–6,205 (65)	6,118–6,180 (63)			TGG
*trn*I	6,205–6,267 (63)	6,181–6,244 (64)			GAT
*trn*K	6,273–6,338 (66)	6,246–6,309 (64)			CTT
*nad*3	6,342–6,689 (348)	6,310–6,657 (348)	ATG/TAA	GTG/TAA	
*trn*S	6,689–6,747 (59)	6,656–6,716 (61)			GCT
*trn*W	6,755–6,820 (66)	6,716–6,778 (63)			TCA
*cox*1	6,824–8,443 (1,620)	6,782–8,401 (1,620)	ATG/TAA	ATG/TAA	
*trn*T	8,429–8,495 (67)	8,387–8,451 (65)			TGT
*rrn*L	8,496–9,468 (973)	8,452–9,412 (961)			
*trn*C	9,469–9,529 (61)	9,418–9,475 (58)			GCA
*rrn*S	9,530–10,266 (737)	9,476–10,200 (725)			
*cox*2	10,267–10,844 (578)	10,201–10,785 (585)	ATG/TAA	ATG/TAA	
*trn*E	10,853–10,920 (68)	10,787–10,853 (67)			TTC
*nad*6	10,923–11,375 (453)	10,855–11,307 (453)	ATG/TAA	ATG/TAG	
*trn*Y	11,379–11,441 (63)	11,314–11,376 (63)			GTA
SNR	11,442–11,508 (67)	11,377–11,441 (65)			
*trn*L	11,509–11,574 (66)	11,443–11,512 (70)			TAG
*trn*S	11,604–11,661 (58)	11,550–11,609 (60)			TGA
*trn*L	11,673–11,738 (66)	11,612–11,680 (69)			TAA
*trn*R	11,744–11,802 (59)	11,680–11,734 (55)			TCG
*nad*5	11,803–13,371 (1,569)	11,729–13,303 (1,575)	GTG/TAA	ATG/TAA	
LNR	13,372–13,522 (151)	13,304–13,747 (444)			

In accordance with other mtDNAs of flatworms sequenced to date (Jia et al., 2010; Liu et al., 2011), the nucleotide compositions are mostly biased toward T, while least favored toward C. AT-richness of mtDNAs in *T. caixuepengi* and *T. tianguangfui* are 71.96% (45.00% T, 26.97% A, 19.17% G, 8.87% C) and 73.48% (46.35% T, 27.13% A, 18.61% G, 7.91% C), respectively.

Flatworms use an unusual mt code to exert protein translation (Nakao et al., 2000; Telford et al., 2000). GTG was used as an alternative initiation codon in *cox*3 and *nad*3 genes of *T. caixuepengi* and *nad*5 gene of *T. tianguangfui*. Furthermore, the codon ATT was inferred as a more unusual start codon of *atp*6 gene in *T. caixuepengi*. The termination codon was mostly TAA, and the ending codon TGA was deprecated (Table 1).

### Morphological Description

**FIGURE 2:**
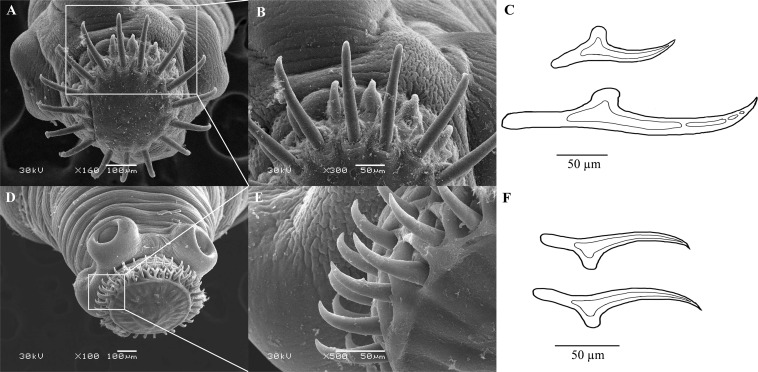
Scanning electron micrographs of scoleces and line drawing of hooks of *Taenia tianguangfui* larva **(A–C)** and *Taenia caixuepengi* larva **(D–F)**.

By gross observation and measurement, *T. caixuepengi* larva was translucent and stripped, 33-36 mm long, and contains cystic fluid (Supplementary Figures S1A,B); *T. tianguangfui* larva on the other hand was opaque, bean-shaped and about 4–10 mm in length (Supplementary Figures S1C,D). Scanning electron microscope observation of their scoleces showed that *T. caixuepengi* larva had four suckers, 16 large hooks and 18 small hooks, the average length of which was about 250 and 100 μm, respectively (Figures 2A–C); *T. tianguangfui* larva also had four suction cups, and 31–33 large and small hooks of about 100 and 80 μm in length, respectively (Figures 2D–F).

### Phylogenetic Relationships

**FIGURE 3:**
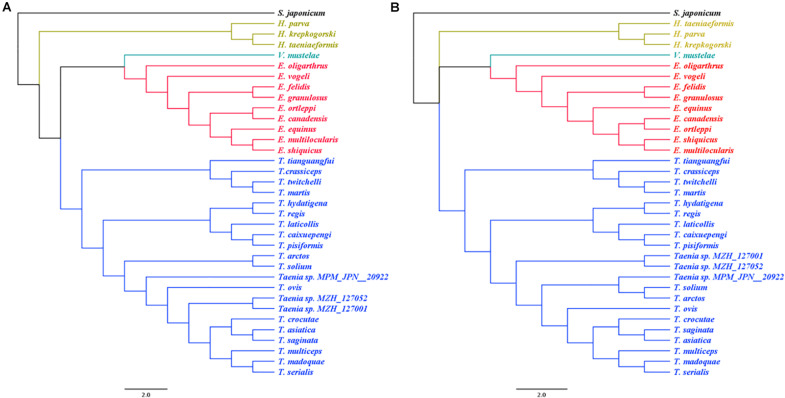
The phylogenetic relationship of *Taenia tianguangfui* and *Taenia caixuepengi*, with other 32 tapeworm species inferred from a Bayesian method based on the concatenated amino acid **(A)** and CDS alignments **(B)** of mitochondrial 12 protein-encoding genes. The species’ name corresponding to the GenBank ID is given in the Supplementary Table S2. The *Echinococcus* spp. are depicted in red, the *Taenia* spp. are depicted in blue, the *Hydatigera* spp. are depicted in yellow and the only one *Versteria* species, *Versteria mustelae*, is depicted in green. The *Schistosoma japonicum* depicted in black was chosen as outgroup.

Phylogenies inferred from both tandem amino acid sequences and DNA sequences of the 12 mt protein-encoding genes demonstrated *T. caixuepengi* in a monophyletic group with *T. pisiformis* and *T. laticollis*, with the closest genetic relative being *T. pisiformis*; *T. tianguangfui* was also found in a monophyletic group with *T. crassiceps*, *T. twitchelli*, and *T. martis*, and has a distant genetic relationship with *T. caixuepengi* (Figure 3).

### Divergence Times Analyses

**FIGURE 4:**
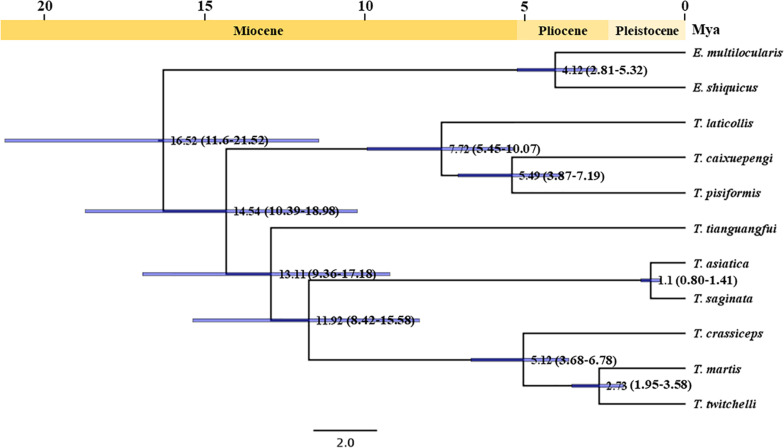
Divergence times construction for *Taenia tianguangfui* and *Taenia caixuepengi* based on the concatenated CDS alignments of mitochondrial 12 protein-encoding genes. The number at the node represents the divergence time between two lineages. The blue bar represents interval of 95% highest probability density, and the number in brackets represents the detailed time interval of 95% highest probability density of divergence time between two lineages. A time scale shows the extent of the Miocene, Pliocene and Pleistocene period.

The divergence time analysis based on mitochondrial protein-coding genes suggested that *T. saginata* and *T. asiatica* should diverge at 1.10 Mya (0.80–1.41, 95% highest probability density) in the early Pleistocene period, which is consistent with the previous reports based on genomic genes (Michelet and Dauga, 2012; Wang et al., 2016); *T. caixuepengi* should diverge from *T. pisiformis* 5.49 Mya (3.87–7.19, 95% highest probability density) in the initial Pliocene period, which is close to the divergence time between *E. shiquicus* and *E. multilocularis* (4.12 Mya, 2.81–5.32, 95% highest probability density); *T. tianguangfui* on the other hand, originated 13.11 Mya (9.36–17.18, 95% highest probability density) in the middle Miocene period, which was earlier than the differentiation of its intermediate host, *N. fuscus* (4.98 Mya, 4.08–5.90, 95% highest probability density) (Figure 4 and Supplementary Figure S2).

## Discussion

The discovery of these two new parasites, *T. caixuepengi* and *T. tianguangfui*, highlights the species diversity of the family Taeniide, and further proved the true biodiversity characteristic of the QTP. Given the lack of human intervention and the rich diversity of wild host species, the present understanding of the species diversity within this family in QTP is apparently just a tip of the iceberg. This is not surprising, given the appreciable cryptic diversity so far uncovered within the taeniid family in Africa and northern latitudes (Lavikainen et al., 2011, 2013; Terefe et al., 2014).

Morphological features traditionally used to distinguish the cysticerci, including the number of hooks, the length of large hooks and small hooks (Loos-Frank, 2000), are insufficient in inferring evolutionary lineages. This is because homoplasy of morphological characters can represent a serious obstacle in taxonomic investigation (Scholz et al., 2020). Here the whole mt genomes of both species were sequenced, and clearly different from all available *Taenia* mt genome sequences, verifying the validity of their species status. Their mt genomes were similar as those of other sequenced tapeworms with respect to length, nucleotide bias, and their tRNA, rRNA and protein-encoding genes composition (Figure 1; Le et al., 2000; Nakao et al., 2003; Jeon et al., 2005; Jeon et al., 2007). Furthermore, the codon ATT was inferred as a more unusual start codon for the *atp*6 gene of *T. caixuepengi* (Table 1), which is a common start codon used by *Caenorhabditis elegans* and *Ascaris suum* (Okimoto et al., 1990).

*T. caixuepengi* larva is so far undetected in other animals, except plateau pika (*O. curzoniae*), meanwhile, no other cysticerci have been found in plateau pika hitherto. Lagomorph is the intermediate host of *T. pisiformis* and *T. laticollis* (Valdmann et al., 2004; Hallal-Calleros et al., 2016). Although similar in appearance and size to the vole, the plateau pika belongs to Lagomorpha (Smith et al., 2019). The close phylogenetic relatedness of these three *Taenia* species (Figure 3) is further highlighted by their high preference for lagomorphs as an intermediate host. Based on the divergence time and phylogeographic analyses, the extent pikas (genus *Ochotona*) originated on the QTP in the middle Miocene, ∼14 Mya (Wang et al., 2020). However, the rapid speciation of many *Ochotona* species, including *O. curzoniae*, occurred during the late Miocene and early Pliocene period (Wang et al., 2020), which almost coincided with the rapid uplift of the QTP (An et al., 2006; Li et al., 2007; Shi et al., 2015). Coincidentally, the evolutionary divergence time analysis in this study also suggests that both *T. caixuepengi* and *E. shiquicus* had evolved in the early Pliocene epoch, about 5.49 and 4.12 Mya, respectively (Figure 4). These almost synchronous events may not have happened by chance. Large-scale diversification of species is often provoked by abiotic factors, such as changes in the living environment and food supply (Benton, 2009). The uplift of the QTP from south to north provided climatic opportunities and food supply for the diversification of cold temperature-preferring pikas but led to the extinction of other warm temperature-preferring rodents (Wang et al., 2020).

For most free-living organisms, speciation is usually the result of genetic drift or adaptive differentiation between geographically separate populations (Turelli et al., 2001). For parasites, however, it has long been thought that sympatric speciation of parasites is common, mediated by ecological isolation caused by host switching within the same geographic region (de Meeûs et al., 1998; Paul, 2002; Huyse et al., 2005). Therefore, we speculate that *T. pisiformi*s in the QTP (Li et al., 2013) may share a common ancestor with *T. caixuepengi*; the split of the pika population caused the ecological isolation between their ancestral populations, which further resulted in the lack of gene flow between them due to intermediate host switching, and the eventual formation of two different species. It can also be speculated that the differentiation pattern between *E. shiquicus* and *E. multilocularis* is similar as that of *T. caixuepengi* and *T. pisiformis*.

Our evolutionary divergence time analysis suggests that the speciation of *T. tianguangfui* occurred in the middle Miocene period (∼13.11 Mya) (Figure 4) when the QTP was undergoing a slow uplift period (An et al., 2006). The timing of the divergence of *N. fuscus* evolved from ∼4.98 Mya (Supplementary Figure S2), which also coincided with the rapid uplift of the QTP (An et al., 2006; Li et al., 2007; Shi et al., 2015). As the species spread in the QTP and Himalaya (Pradhan et al., 2019), the evolutionary origin of the *Neodon* spp., like the plateau pika, may well be due to changes in climate and food supply caused by the uplift of the QTP and Himalaya. The speciation of *T. tianguangfui* was earlier than that of its intermediate host, indicating that *T. tianguangfui* did not differentiate into a new *Taenia* species in order to adapt to the intermediate host, rather, it suggests that *T. tianguangfui* larva might not be limited to *N. fuscus*. *Taenia crassiceps* and *T. martis* have similar intermediate hosts range, infecting a variety of rodents, even humans and other primates (Deplazes et al., 2019). Given the close relationship between *T. tianguangfui*, *T. crassiceps*, and *T. martis*, it also cannot be excluded that *T. tianguangfui* may be infective to a variety of rodents other than *N. fuscus*, as well as humans and other primates. So far, a clear understanding of their evolutionary origin from these clues is elusive, thus, more data and investigation are needed to provide further insight.

Adult worms of the *T. tianguangfui* and *T. caixuepengi* have not yet been collected due to the difficulty in sampling endangered or protected carnivores. However, nucleic acid from feces of wolves, foxes and dogs found at the sampling sites were examined, so far, no positive feces samples for these two species have been found. Plateau pikas and voles are the primary food source for wild canids across the QTP. Tibetan foxes are the obligate predator of plateau pikas, as their remains (plateau pikas) are often encountered in 99% of their feces (Smith et al., 2019). Wild canids, especially the red fox and the Tibetan fox, may well be important definitive hosts for *T. tianguangfui* and *T. caixuepengi*.

Adult or larval samples of tapeworm are easily damaged in the process of collection, freeze-thaw and processing, and the morphological features are mostly unidentifiable (Lavikainen et al., 2013). While mt genome data alone may not fully answer the scientific questions surrounding their evolutionary origins, it is the most cost-effective and accurate method. Recently, although laborious and costly, there have been an increasing whole genome sequencing and analyses for many tapeworm species. This kind of investigation, not only is it important to provide insights into their host adaptation and switching, evolution mechanisms through gene groups amplification, hosts-parasites interaction, immune regulation and nutrition, it also provides urgently needed resources for the identification of drug target and diagnostic molecular markers (Wang et al., 2016; International Helminth and Genomes Consortium, 2019). In the future, a lot of genomic data will be needed to study this fascinating group.

In conclusion, the mitochondrial genome sequence data adequately confirm the validity of the two new *Taenia* species named *T. caixuepengi* and *T. tianguangfui*, we have previously reported. The phylogenetic trees and divergence times analyses suggest that *T. caixuepengi* evolve from its closest relative, *T. pisiformis*, in the initial Pliocene period (∼5.49 Mya), due to the intermediate host switching caused by the rapid uplift of the QTP; *T. tianguangfui* is probably parasitic in a wide variety of rodents, and share a common ancestor with *T. crassiceps*, *T. twitchelli* and *T. martis*, splitting in middle Miocene period (∼13.11 Mya).

## Data Availability Statement

The datasets presented in this study can be found in online repositories. The names of the repository/repositories and accession number(s) can be found below: https://www.ncbi.nlm.nih.gov/nucleotide.. GenBank ID: MT882036 and MT882037.

## Ethics Statement

The animal study was reviewed and approved by the Animal Ethics Procedures and Guidelines of the People’s Republic of China and Animal Ethics Committee of Lanzhou Veterinary Research Institute, Chinese Academy of Agricultural Sciences (No. LVRIAEC2012-007).

## Author Contributions

Y-DW, LL, H-BY, and W-ZJ conceived and designed the experiments. Y-DW, Y-LF, and X-WN performed the experiments. Y-DW and Y-LF performed the data analyses. Y-DW prepared the figures and wrote the manuscript. JO provided improving paragraphs and language editing. W-HL, N-ZZ, and B-QF provided very constructive suggestions for revisions. All authors read and approved the final manuscript.

## Conflict of Interest

The authors declare that the research was conducted in the absence of any commercial or financial relationships that could be construed as a potential conflict of interest.

## Footnotes

1 https://www.ncbi.nlm.nih.gov/genbank/

2 https://chlorobox.mpimp-golm.mpg.de/geseq.html

3 http://130.235.46.10/ARWEN/

## Supplementary Material

Supplementary Figure 1 The larvae of *Taenia caixuepengi* and *Taenia tianguangfui* were found in Plateau pikas *(Ochotona curzoniae)* and Qinghai voles (*Neodon fuscus*), respectively. The banded larvae of *T. caixuepengi* in the plate **(A)** were picked out from the enterocoelia of Plateau pikas **(B)**, and the granular larvae of *T. tianguangfui* in the plate **(C)** were picked out from the enterocoelia and chest of Qinghai voles **(D)**. The blue arrow indicates the parasitic larvae.

Supplementary Figure 2 Divergence times construction for *Neodon fuscus* based on the concatenated CDS alignments of mitochondrial 13 protein-encoding genes. The number at the node represents the divergence time between two lineages. The blue bar represents interval of 95% highest probability density, and the number in brackets represents the detailed time interval of 95% highest probability density of divergence time between two lineages. A time scale shows the extent of the Miocene, Pliocene and Pleistocene period.

Supplementary Table 1 Detailed sample collection data.

Supplementary Table 2 The primers for amplifying the complete mitochondrial genomes of cysticercus.

Supplementary Table 3 GenBank accession numbers of mitochondrial genome sequences used for phylogenetic analyses and divergence times analyses in this study.
