# Supplementary material for: Genetic Evolution and Implications of the Mitochondrial Genomes of Two Newly Identified Taenia spp. in Rodents From Qinghai-Tibet Plateau
Source: Front Microbiol. 2021 Mar 23;12:647119. doi: 10.3389/fmicb.2021.647119 (PMC8021716; doi:10.3389/fmicb.2021.647119)
Supplement: Supplementary Figure 1 — The larvae of Taenia caixuepengi and Taenia tianguangfui were found in Plateau pikas (Ochotona curzoniae) and Qinghai voles (Neodon fuscus), respectively. The banded larvae of T. caixuepengi in the plate (A) were picked out from the enterocoelia of Plateau pikas (B), and the granular larvae of T. tianguangfui in the plate (C) were picked out from the enterocoelia and chest of Qinghai voles (D). The blue arrow indicates the parasitic larvae. [file Data_Sheet_1.docx]

Table S2 | The primers for amplifying the complete mitochondrial genomes of cysticercus

| Primer ID | Sequence (5’-3’) |
| --- | --- |
| F1 | CARTTRTTRAGWCAGGGBGTTTCTAAGTG |
| R1 | AAATCCWACWTTYCADACACCCTTCTT |
| F2 | TAAACTRRTAGATTGTGGTTCTRTTGAATACT |
| R2 | AAAAYGCYAARCAACGCTTMCCATC |
| F3 | GTGARTCTCCDTATTCTGAGCG |
| R3 | AAAAAACARAARAATAWWACYGGAAACTTCAT |
| F4 | AGTTTGGDTTRTTTCCDTTTRTGTT |
| R4 | TGACACRAAATTATTAGCAGTAACTYCACA |
| F5 | CGTGATGCTGTTAACTTCARGAAATGG |
| R5 | CCYAAACADACTATWGAAAACATAGC |
| F6 | TGATCGTAAATTTAGWTCWGCATTTTTTGATC |
| R6 | TCACGTCAAACCATTCARACAAGCC |
| F7 | TTATTTCTCAGGGTCTTTCCGTCTGTTTA |
| R7 | CTTCAAATTCATTTAAAGTTACCTTGTTACG |
| F8 | TGACAGTGATTAGATACCCCATTA |
| R8 | TTYCTTTTGGCCGTAACATAAG |
| F9 | ATATGAGTTAGTTTTAAGCATTAATTATGG |
| R9 | TGCTTAGTAAAAAAYACTCCWATAAATGG |

Table S3 | GenBank accession numbers of mitochondrial genome sequences used for phylogenetic analyses and divergence times analyses in this study

| Family | Genus | Species | GenBank accession no. |
| --- | --- | --- | --- |
| Taeniidae | *Echinococcus* | *Echinococcus canadensis* | NC_011121 |
|  |  | *Echinococcus equinus* | NC_020374 |
|  |  | *Echinococcus felidis* | NC_021144 |
|  |  | *Echinococcus granulosus* | NC_044548 |
|  |  | *Echinococcus multilocularis* | NC_000928 |
|  |  | *Echinococcus oligarthrus* | NC_009461 |
|  |  | *Echinococcus ortleppi* | NC_011122 |
|  |  | *Echinococcus shiquicus* | NC_009460 |
|  |  | *Echinococcus vogeli* | NC_009462 |
|  | *Hydatigera* | *Hydatigera krepkogorski* | NC_021142 |
|  |  | *Hydatigera parva* | NC_021141 |
|  |  | *Hydatigera taeniaeformis* | NC_014768 |
|  | *Taenia* | *Taenia caixuepengi* | MT882036 |
|  |  | *Taenia tianguangfui* | MT882037 |
|  |  | *Taenia arctos* | NC_024590 |
|  |  | *Taenia asiatica* | NC_ 004826 |
|  |  | *Taenia crassiceps* | NC_002547 |
|  |  | *Taenia crocutae* | NC_024591 |
|  |  | *Taenia hydatigena* | NC_012896 |
|  |  | *Taenia laticollis* | NC_021140 |
|  |  | *Taenia madoquae* | NC_021139 |
|  |  | *Taenia martis* | NC_020153 |
|  |  | *Taenia multiceps* | NC_012894 |
|  |  | *Taenia ovis* | NC_021138 |
|  |  | *Taenia pisiformis* | NC_013844 |
|  |  | *Taenia regis* | NC_024589 |
|  |  | *Taenia serialis* | NC_021457 |
|  |  | *Taenia saginata* | NC_009938 |
|  |  | *Taenia solium* | NC_004022 |
|  |  | *Taenia twitchelli* | NC_021093 |
|  |  | *Taenia sp. MPM_JPN__20922* | AB905202 |
|  |  | *Taenia sp. MZH_127001* | AB905200 |
|  |  | *Taenia sp. MZH_127052* | AB905203 |
|  | *Versteria* | *Versteria mustelae* | NC_021143 |
| Schistosomatidae | *Schistosoma* | *Schistosoma japonicum* | NC_002544 |
| Cricetidae | *Lasiopodomys* | *Lasiopodomys mandarinus* | NC_025283 |
|  | *Microtus* | *Microtus fortis calamorum* | NC_015243 |
|  |  | *Microtus fortis fortis* | NC_015241 |
|  |  | *Microtus kikuchii* | NC_003041 |
|  | *Neodon* | *Neodon fuscus* | NC_040138 |
|  |  | *Neodon irene* | NC_016055 |
|  |  | *Neodon sikimensis* | NC_035503 |
| Muridae | *Mus* | *Mus musculus* | NC_005089 |
|  | *Rattus* | *Rattus norvegicus* | NC_001665 |


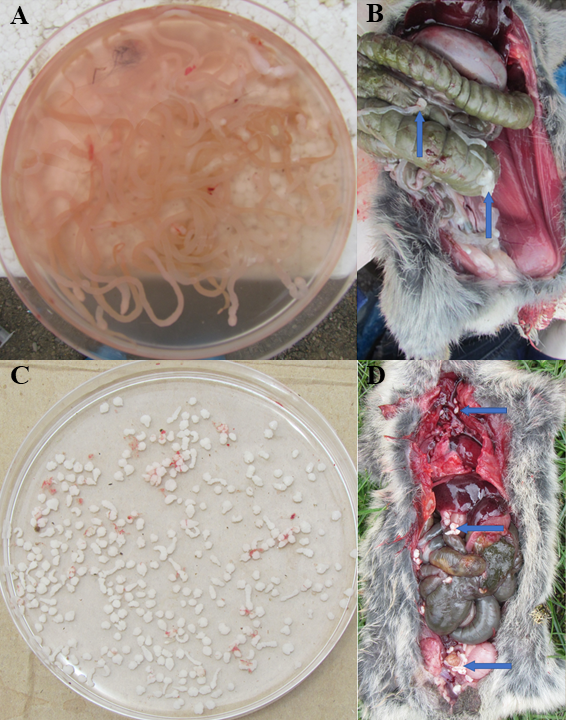
Figure S1 | The larvae of *Taenia caixuepengi* and *Taenia tianguangfui* were found in Plateau pikas (*Ochotona curzoniae*) and Qinghai voles *(Neodon fuscus*), respectively


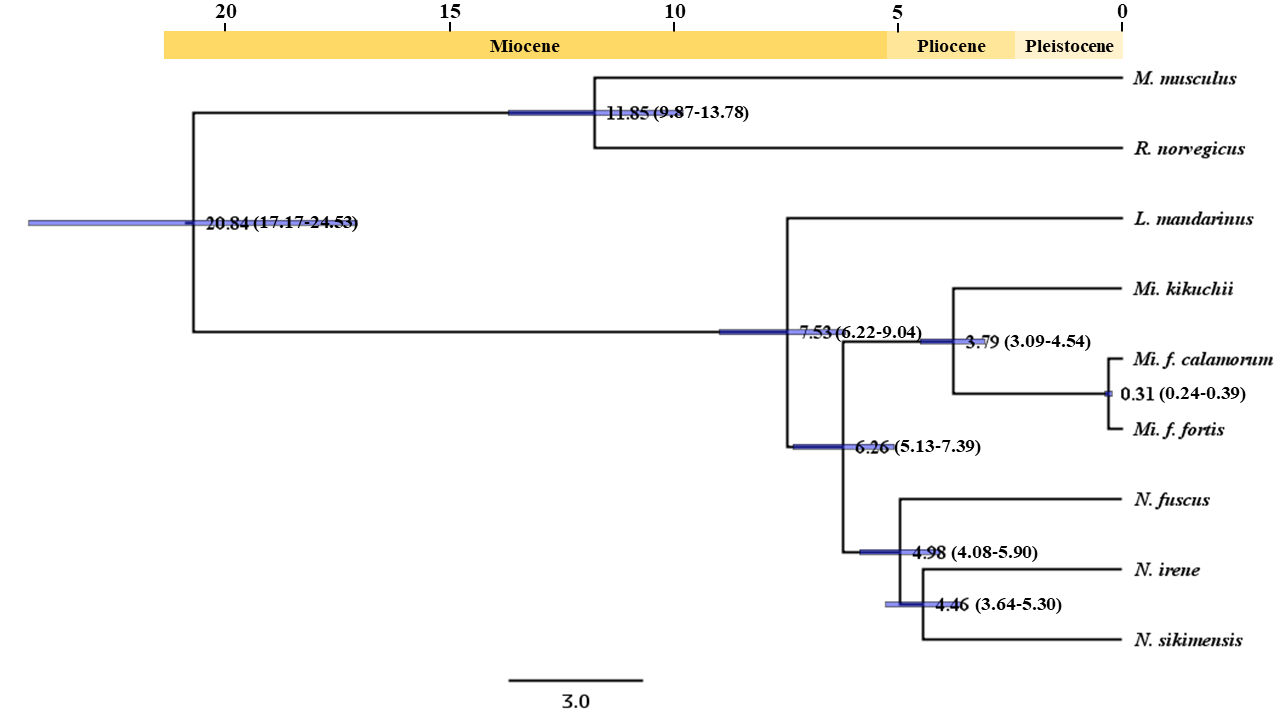
Figure S2 | Divergence times construction for *Neodon fuscus* based on the concatenated CDS alignments of mitochondrial 13 protein-encoding genes
